# Etiology of Severe Pneumonia in Children in Alveolar Lavage Fluid Using a High-Throughput Gene Targeted Amplicon Sequencing Assay

**DOI:** 10.3389/fped.2021.659164

**Published:** 2021-06-25

**Authors:** Fei Li, Yin Wang, Yuhan Zhang, Peng Shi, Linfeng Cao, LiYun Su, Qiguo Zhu, Libo Wang, Roujian Lu, Wenjie Tan, Jun Shen

**Affiliations:** ^1^Infectious Disease Department, Children's Hospital of Fudan University, National Children's Medical Center, Shanghai, China; ^2^Clinical Trial Unit, Children's Hospital of Fudan University, National Children's Medical Center, Shanghai, China; ^3^Statistics and Data Management Center, Children's Hospital of Fudan University, National Children's Medical Center, Shanghai, China; ^4^Virology Department, Children's Hospital of Fudan University, National Children's Medical Center, Shanghai, China; ^5^Respiratory Department, Children's Hospital Xiamen Branch, Xiamen, China; ^6^Respiratory Department, Children's Hospital of Fudan University, National Children's Medical Center, Shanghai, China; ^7^National Institute for Viral Disease Control and Prevention, China CDC, Beijing, China

**Keywords:** high-throughput sequencing, targeted amplicon sequencing, multiplex polymerase chain reaction, severe community-acquired pneumonia, alveolar lavage fluid, children

## Abstract

**Objective:** To evaluate the diagnostic value of a high-throughput gene targeted amplicon sequencing (TAS) assay for detecting pathogenic microorganisms in alveolar lavage fluid (ALF) from children with severe community-acquired pneumonia (SCAP).

**Methods:** A retrospective study was performed on 48 frozen ALF samples from 47 severe pneumonia cases admitted to Children's Hospital of Fudan University from January 1, 2019, to March 31, 2019. All samples were tested by a multiplex PCR (Multi-PCR) assay and a TAS assay. The results of the TAS panels were parallel compared with Multi-PCR and Conventional Tests (CT) including culture, direct fluorescent antibody method (DFA), and singleplex polymerase chain reaction (PCR).

**Results:** The proportion of pathogens detection by CT was 81.2% (39/48). The 8 common respiratory viruses including respiratory syncytial virus (RSV), adenovirus (ADV), influenza A virus (FLUA), influenza B virus (FLUB), parainfluenza virus 1–3 (PIV1-3), and human Metapneumovirus (hMPV) were found in 31.2% (15/48) of the 48 samples by DFA. With the criteria of CT results used as “Golden Standard” for determing of TAS results, the proportion of pathogens detection by TAS was 70.8% (34/48). The difference of proportion of pathogens detection between TAS and CT was not statistically significant (*p* = 0.232). The sensitivity and specificity of TAS for pathogens detection based on CT were 87.1% (95% CI, 71.77–95.18%) and 100.0% (95% CI, 62.88–100%), the positive predictive value (PPV) and negative predictive value (NPV) were 100.0% (95% CI, 87.35–100%) and 64.2% (95% CI, 35.62–86.02%), respectively. While Multi-PCR results were used as “Golden Standard,” the total pathogens detection rate of TAS was 83.3% (40/48), which had a significant difference with that of Multi-PCR (*p* = 0.003). The sensitivity and PPV of TAS compared with Multi-PCR were 83.3% (95% CI, 69.23–92.03%) and 100.0% (95% CI, 89.08–100%), respectively. High rates of co-infection were proved by CT, Multi-PCR, and TAS. Mycoplasma pneumoniae (MP) and ADV were the two most frequently detected pathogens in all three assays.

**Conclusion:** Compared with the CT and Multi-PCR methods, this TAS assay had a good performance in detecting bacteriological and viral pathogens from ALF. More research is needed to establish interpretation criteria based on TAS reads or analysis platforms.

## Introduction

Severe pulmonary infection in children is still one of the main causes of infection-related deaths in children (1–3). The Golden Standard for the etiological diagnosis of pulmonary infection is the conventional culture depended diagnositic tests (CDTs). Nasopharyngeal swabs and sputum collected from the upper respiratory tract were commonly used for bacteriological culture, and occasionally, ALF, lung puncture fluid, and tracheal intubation fluid, which are more reflective of lower respiratory tract inflammation, are sent to the culture bench (2, 4, 5). Mainly due to the low positive rate of culture, antigen-based and nucleic acid-based tests, such as DFA and PCR, are often used in clinical settings as “alternative Golden Standard” for etiological diagnosis. In the past decade, the development of culture-independent diagnositic tests (CIDTs), such as serology, antigen detection, PCR, TAS, and metagenomic sequencing (mNGS), had helped the clinical practitioners upgrade understanding of pulmonary infection pathogens and pulmonary microbiome (6–10). mNGS based workflows can detect almost all the nucleic acids of pathogenic microorganisms in specimens, but some shortcomings limit this complex technology from being widely used in clinical. Not only the challenges associated with analytical performance, but also sample preparation and sequencing methodologies should be standardized. The presence of contaminants and host DNA poses big challenges to data analysis. The high sensitivity and specificity of PCR technology can be exploited by designing targeted Multi-PCR systems and TAS based on the presupposition of possible pathogenic agents. It is reasonable to believe that TAS and mNGS will be widely used in the clinic as pathogens detection methods in the next decade. But before that, Multi-PCR might popular for some time (7–9, 11). In another point, children are the most vulnerable to respiratory infections. It is a critical period for the human population to establish herd immunity to normally circulating respiratory pathogens in the early stages of life. But some individuals unfortunately result in severe sequelae and even death from severe pulmonary infections, although the vaccine strategies reduced most of the tragedies nowadays. In this study we tried to use a TAS assay in pediatric clinical to the diagnosis of SCAP etiologies, and map the pathogens of SCAP in children by TAS compared with CT and Multi-PCR to facilitate pediatricians to improve the etiological diagnosis and treatment strategies.

## Materials and Methods

### Study Patients

A retrospective study was conducted on 48 ALF stored samples from 47 children who experienced fiberoptic bronchoscope operation from January to March 2019 in Children's Hospital of Fudan University were obtained (Supplementary Material 1). Patients were included as following: (i) <18 years old; (ii) diagnosis with SCAP according to the diagnostic criteria recommended by the 2011 IDSA CAP management guideline (12); (iii) fiberoptic bronchoscopy was performed and adequate samples of ALF (≥0.5 ml) were preserved. Patients without infectious pneumonia, nosocomial acquired pneumonia, aspiration pneumonia, including airway foreign body inhalation, were excluded. Samples without enough volume (<0.5 ml) to complete Multi-PCR and TAS simultaneously were also excluded.

### Sample Acquisition and Processing

**Figure 1:**
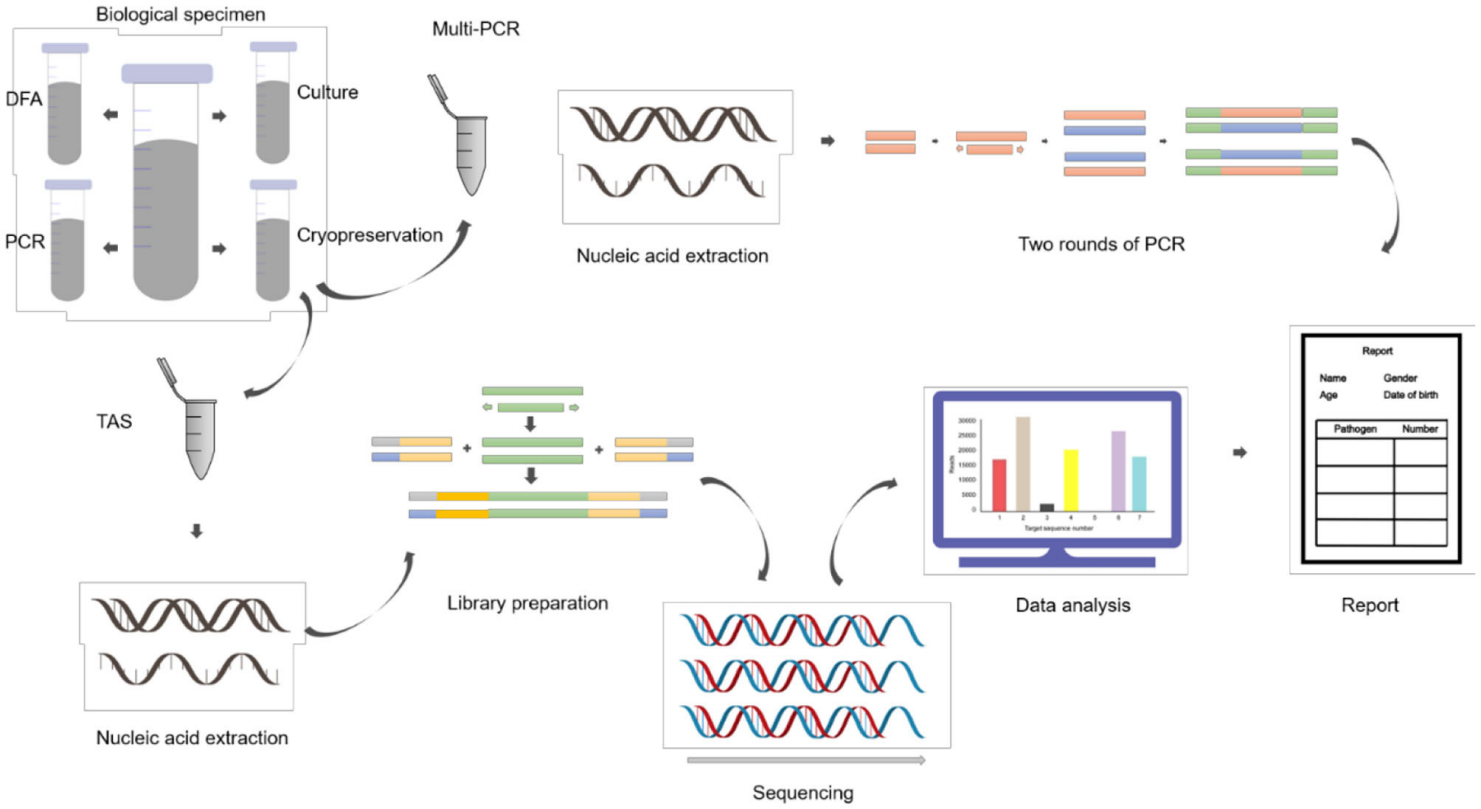
Alveolar lavage fluid (ALF) samples analysis workflow.

ALF samples were collected during fiberoptic bronchoscopy lavage treatment and divided into four parts: three were sent to clinical laboratories for routine tests, each one aliquot for gram staining and bacteriological culture, direct immunofluorescence antigen detection, conventional reverse transcription polymerase chain reaction (RT-PCR) and real-time fluorescent quantitative polymerase chain reaction (qPCR); and one sample was stored in sterile containers frozen at −80°C (Figure 1).

### Clinical Data Collection

Clinical data were obtained from the hospital information system. Two senior infectious diseases (ID) doctors were designated to collect clinical information of the patient, including age, clinical manifestations, conventional test results, imaging findings, diagnosis, and treatment by unified questionnaires.

### Identification of Pathogens

#### Pathogens Identified by CT Methods

All the ALF samples were sent to routine bacteriological culture. Antigen-based testing for target viruses in ALF samples were screened by two commercial DFA assay, as for RSV, ADV, FLUA, FLUB, and PIV1-3 by one DFA assay (Chemicon International Inc., Temecula, CA, USA), and hMPV were screened by another DFA assay (Diagnosis Hybrids Inc., USA). Nucleic acid-based test the ALF samples were complete in the clinical microbiology laboratory. Rhinovirus (RV) was detected by real-time RT-PCR assay (Hubei Langde Medical Technology Co. LTD, China), and MP was detected by a fluorescent real-time PCR assay. Some cases completed a T-cell spot test (T-SPOT) for excluded tuberculosis infection due to their radiographic manifestations of pulmonary infection.

#### Pathogens Identified by Multiplex PCR

RNA/DNA were extracted by QIAamp MinElute Virus Spin Kit (QIAgen, Hilden, Germany) for Multi-PCR. Multi-PCR was performed by LightCycler®480 PCR instrument (Roche, Basel, Switzerland) as follows: 50°C for 10 min; 95°C for 2 min; 40 cycles of 94°C for 20 s, 55°C for 20 s, and 72°C for 35 s; 95°C for 2 min; 10 cycles of 94°C for 20 s, 55°C for 15 s, and 72°C for 15 s; 23 cycles of 94°C for 15 s, 50°C for 15 s, and 72°C for 15 s; 95°C for 2 min; 40°C for 90 s; 95°C for 2 min; 37°C for 1 s. RespiFinder 2SMART multiplex PCR kit for 22 respiratory pathogens including coronavirus and bocavirus was supplied by GeneoDx Biotech Co, Shanghai, China (Supplementary Material 2).

#### Pathogens Identified by High-Throughput Gene Targeted Amplicon Sequencing

The high-throughput gene TAS targeted 412 pathogenic microorganisms (229 bacteria, 51 fungi, 72 viruses, 39 parasites, 21 others) was supplied by U JIA Medical Technology Co., Shanghai, China (Supplementary Material 3).

#### Samples Pretreatment and Nucleic Acid Extraction

Each ALF sample was centrifuged at 13,000 RPM for 10 min in a 2.5 ml centrifuge tube. Discarding the upper part of the liquid, 500 μL sample size remained. The remaining samples were mixed and put into the module of ultrasonic cell fragmentation instrument (JIN XIN, Shanghai), with the frequency of 500 W, 30 min. Ultrasonic operation steps were 4°C, 10 min interval, 1 min for each operation. Then the samples were transferred to the cracking tubes with pre-installed glass beads. The tubes were fixed on the adapter of the grinding instrument (JIN XIN, Shanghai), with 70 HZ, 5 min. The ground samples were used for DNA/RNA extraction and purification (ZymoBIOMICS DNA/RNA Miniprep Kit, R2002).

### Library Construction

Total RNA was subjected to reverse transcription reaction to obtain complementary cDNA, which was used as a template for the construction of pathogens targeted high-throughput sequencing library. The library was built by using Pathogeno One High-throughput sequencing document Building kit (Bingyuan Medical Technology Co., LTD, Shanghai, China), including two rounds of PCR. The first round used the samples nucleic acid and cDNA as templates, and more than 500 microorganisms specific primers were amplified to enrich the pathogens target sequences. The procedure of the first round of PCR was: 95°C for 3 min; 25 cycles of 95°C for 20 s, 60°C for 4 min; 72°C for 5 min. The purpose of the second round of PCR was to purify the products as the template by using magnetic beads. Sequencing adapters and primer with different Barcode were added to amplify, with the procedure as follows: 95°C for 3 min; 8 cycles of 95°C for 15 s, 60°C for 15 s, 72°C for 1 min; 72°C for 5 min. Agarose gel electrophoresis and Qubit4.0 fluorometer were used for quality control and quantification. The normal library fragment size is about 400 bp, and concentration should high than 1 ng/μL (Supplementary Material 4).

#### High-Throughput Gene Targeted Amplicon Sequencing

The libraries of each sample were mixed and diluted to a final concentration of 4 nM. A 5 μl mixture was added with 5 μL of 0.2 mol/L NaOH, then centrifuged at 280 g for 1 min. Followed with vortex oscillation, the samples were exposed at room temperature 5 min for denaturation. An Illumina MiSeq Reagent Nano Kit was used for high-throughput sequencing by the platform of Illumina MiSeq. The average amount of reads was 0.03~0.05 m per library, and the sequencing read length was PE60.

#### Data Analysis of TAS

The original data of deactivation were first used to identify the adaptor. Identified reads of the adaptor and subsequent sequences were cut off. Reads with the length of both ends >60 bp were retained. Low quality filtering was then performed, reads Q30 > 50% were retained as high-quality data, which identified by the primers. Reads with correct double-ended alignment were retained, and compared with the pathogens database to reconfirm the pathogens types.

### Criteria for a Positive TAS Result

(i) Organisms were known to be potentially pathogenic; (ii) Pathogens were both identified by CT and TAS; (iii) Pathogens were both identified by Multi-PCR and TAS.

### Statistical Analysis

SPSS 23.0 statistical software was used for statistical analysis and a two-tailed value of p of 0.05 was considered for significant differences. Continuous variables with normal distribution were expressed as mean ± standard deviation (SD), whereas continuous data with non-normal distribution were expressed as median (IQR, inter-quartile range) and categorical data were expressed as numbers (percentage).

## Results

### Demographic Characteristics

The clinical features of the 47 patients with SCAP, including age and gender, underlying diseases, white blood cells (WBC) count and differential white cell counts, C-reactive protein (CRP), and treatment are presented in Table 1. One case stayed in hospital only 1 day and then referred to the outpatient department. T-SPOT test was ordered in 31 cases and no positive cases were found. One patient had experienced a second fiberscope intervention 3 days later because his lung consolidation failed to improve after the first fiberscope intervention.

**Table 1:** Clinical characteristics of patients.

**Characteristics**	**Patients (*n* = 47)**
Age, years, median (Q1, Q3)	6.0 (2.7, 8.0)
Gender, female, *n* (%)	24 (51.1)
Basic diseases, *n* (%)	4 (8.5)
Immunodeficiency disease, *n* (%)	2 (4.3)
Epilepsy, *n* (%)	1 (2.1)
Chronic diarrhea with pancreatic exocrine dysfunction, *n* (%)	1 (2.1)
**Laboratory examination**	
White blood cells (X10*9), mean ± SD	8.87 ± 3.69
Neutrophils (%), median (Q1, Q3)	60.0 (40.0, 69.0)
Lymphocyte (%), median (Q1, Q3)	31.3 (22.1, 51.2)
C-reactive protein (mg/dl), median (Q1, Q3)	0 (0, 36.0)
Application of antibiotics, *n* (%)	47 (100.0)
Application of antifungal agent, *n* (%)	3 (6.4)
Application of antivirals, *n* (%)	4 (8.5)
Hospital stays, days, median (Q1, Q3)	6.0 (1.0, 9.0)
Treatment in ICU, *n* (%)	1 (2.1)
Support of mechanical ventilation, *n* (%)	0 (0.0)

### TAS Positive Pathogens Identification Based on CT Results

**Figure 2:**
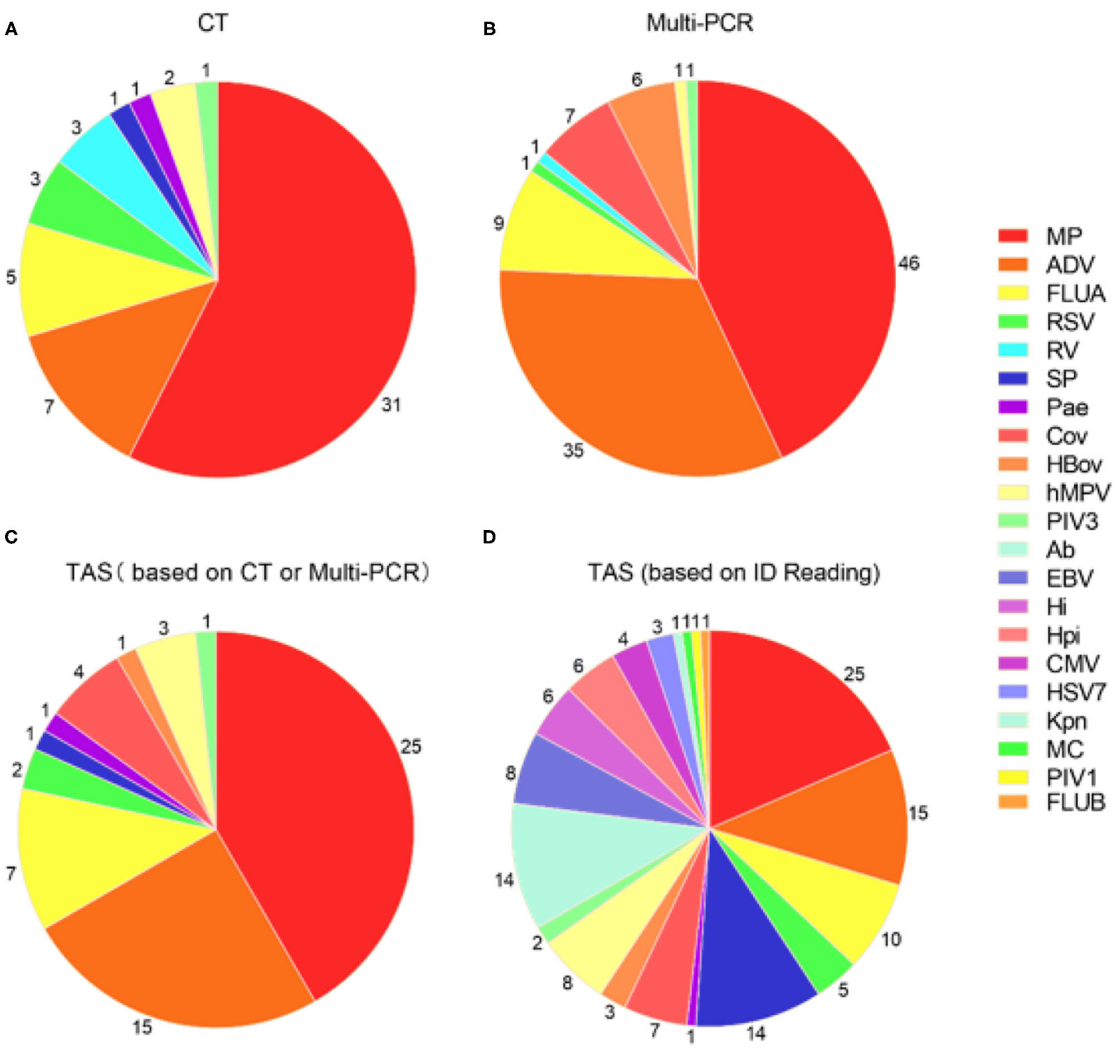
Pathogens detection in 48 samples by three different detection methods. **(A)** Pathogens detected by CT; **(B)** Pathogens detected by Multi-PCR; **(C)** TAS pathogens detection based on CT or Multi-PCR; **(D)** TAS pathogens detection based on the infectious diseases doctors' judgment. MP, *Mycoplasma pneumoniae*; ADV: Adenovirus; SP, *Streptococcus pneumonia*; Ab, *Acinetobacter baumannii*; FLUA, Influenza A virus; hMPV, human Metapneumovirus; EBV, Epstein-barr virus; Hi, *Haemophilus influenzae*; Hpi, *Hemophilus parainfluenzae*; OC43, Coronavirus OC43; RSV, Respiratory syncytial virus; CMV, Cytomegalovirus; HBov, Human bocavirus; HSV7, Human herpesvirus type 7; 229E, Coronavirus 229E; PIV3, Parainfluenza virus type 3; Pae, *Pseudomonas aeruginosa*; Kpn, *Klebsiella pneumoniae*; MC, *Moraxella catarrhalis*; PIV1, Parainfluenza virus type 1; FLUB, Influenza B virus; HKU1, Coronavirus HKU1; NL63, Coronavirus NL63.

**Figure 3:**
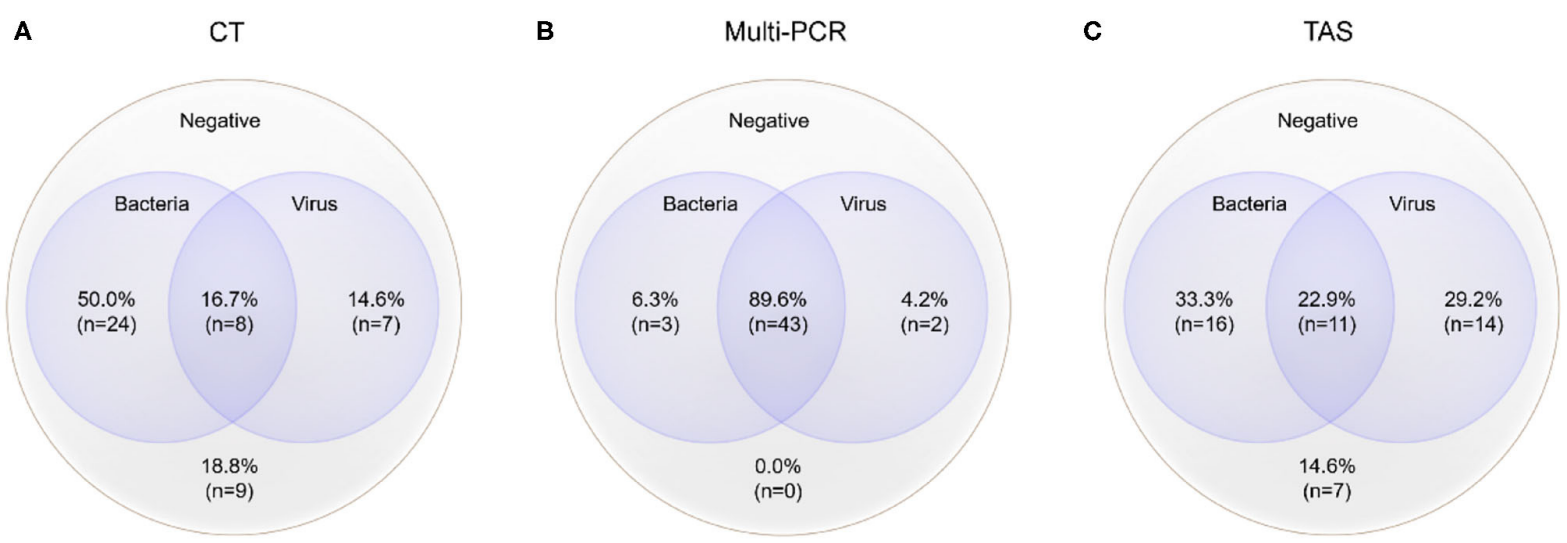
Mixed pathogens detection by three different methods (MP was counted as atypical bacteria); the cross-section refers to the co-detection of viruses and bacteria, excluding the co-detection of different viruses in the same sample. **(A)** Pathogens detected by CT; **(B)** Pathogens detected by Multi-PCR; **(C)** Pathogens detected by TAS.

The total pathogens detection rate by CT was 81.2% (39/48) in the 48 specimens, which including the results of DFA, singleplex PCR, and culture (Figure 2). MP was positive in both specimens from the case with two fiberscope intervention. Mixed pathogens were detected in 22.9% (11/48) of the samples (Figure 3). The positive rates of MP, ADV, FLUA, RSV,RV, RSV, hMPV, and PIV3 were 64.5% (31/48), 14.5% (7/48), 10.4% (5/48), 6.2% (3/48), 6.2% (3/48), 6.2% (3/48), 4.1% (2/48), and 2.0% (1/48), respectively. MP and ADV were the two dominant pathogens. Eight common respiratory (RSV, ADV, FLUA/B, PIV1-3, and hMPV) viruses were found in 31.2% (15/48) of the 48 samples by DFA. One strain of *Pseudomonas aeruginosa* (Pae) and one *Streptococcus pneumoniae* (SP) were isolated in two samples by bacteriological culture (Table 2).

**Table 2:** Pathogens detection in 48 samples by their methods.

	**Viruses**, ***n*** **(TAS reads range)**										**MP *n* (TAS reads range)**	**Bacteria *n* (TAS reads)**	**Co-detection**
	**ADV**	**FLUA**	**RSV**	**PIV3**	**hMPV**	**RV**	**HBov**	**OC43**	**229E**	**NL63/HKU1**			
CT positive	7	5	3	1	2	3	/	/	/	/	31	2	11
Multi-PCR positive	35	9	1	1	1	1	6	4	2	1	46		45
TAS positive based on CT	6 (140–6,574)	3 (41–5,125)	2 (1–1,083)	1 (7,174)	1 (1,251)	0	/	/	/	/	23 (4–24,153)	2 (3,223–27,926)	4
TAS positive based on Multi-PCR	13 (7–6,574)	7 (41–9,456)	1 (1,083)	1 (7,174)	1 (10,956)	0	1 (6)	4 (7–6,716)	0	0	25 (4–24,253)		12
TAS positive based on CT or Multi-PCR	14 (7–6,574)	7 (41–9,456)	2 (1–1,083)	1 (7,174)	2 (1,251–10,956)	0	1 (6)	4 (7–6,716)	0	0	25 (4–24,253)	2 (3,223–27,926)	17

With the criteria for a positive TAS result both identified by CT, or CT results were used as “Golden Standard” for determining of TAS results, the total pathogens detection rate was 70.8% (34/48) ignoring some low reads number. There was no significant differences of total pathogens detection rate between TAS and CT (*p* = 0.232). In this TAS assay, no primers were targeted to PIV2 (Supplementary Material 3). The seven common respiratory viruses (ADV, FLUA, RSV, RSV, hMPV, PIV1, and PIV3) were found in 25.0% (12/48) of samples. MP and ADV were also the two dominant pathogens in those samples. TAS method also successfully found the two bacteria in two samples. The sensitivity and specificity of TAS for pathogens detection were 87.1% (95% CI, 71.77–95.18%) and 100.0% (95% CI, 62.88–100%) (Table 2).

### TAS Positive Pathogens Identification Based on Multi-PCR Results

With the criteria for a positive TAS result both identified by Multi-PCR, or Multi-PCR results were used as “Golden Standard” for determining of TAS results, the total pathogens detection rate was 83.3% (40/48). The two samples from the same case were both positive with MP by Multi-PCR and TAS. Significant differences was found in total pathogens detection rate between TAS and Multi-PCR (*p* = 0.003). The sensitivity and PPV of TAS compared with Multi-PCR were 83.3% (95% CI, 69.23–92.03%) and 100.0% (95% CI, 89.08–100%), respectively. The eight common respiratory viruses were found in 45.8% (22/48) of samples. MP and ADV were also the two dominant pathogens in those samples by TAS. Those two dominant pathogens had a high positive rate of 52.0% (25/48) and 31.2% (15/48), respectively, but lower than the results of 95.8% (46/48) and 72.9% (35/48) from Multi-PCR. In this TAS assay, no primers were targeted to PIV2 (Supplementary Material 3). One sample was found HBov positive by TAS assay, while six samples positive by Multi-PCR. Four common coronavirus (OC43, 229E, HKU1, and NL63) were found in seven samples by Multi-PCR method, and 4 strains of OC43 were confirmed by TAS (Figure 2).

### TAS Positive Pathogens Identification Based on CT or Multi-PCR Results

With the criteria for a positive TAS result based on any of the other two assay positive reports, the total pathogens detection rate of TAS was 85.4% (41/48) and had significant difference in total pathogens detection rate between TAS (41/48) and CT (39/48), *p* = 0.584. Four CT negative samples found had positive pathogens in TAS, when TAS combined with Multi-PCR results. Meanwhile, each one strain of RSV, hMPV, and Pae were additionally isolated from three of the 48 Multi-PCR positive samples (Table 3).

**Table 3:** TAS positive pathogens identification based on CT or Multi-PCR results.

	**CT+**	**CT–**	**Multi-PCR+**
TAS+	37	4 (ADV, FLUA, MP, MP + ADV)	41 (plus RSV, hMPV and Pae)
TAS–	2	5	8

**Figure 4:**
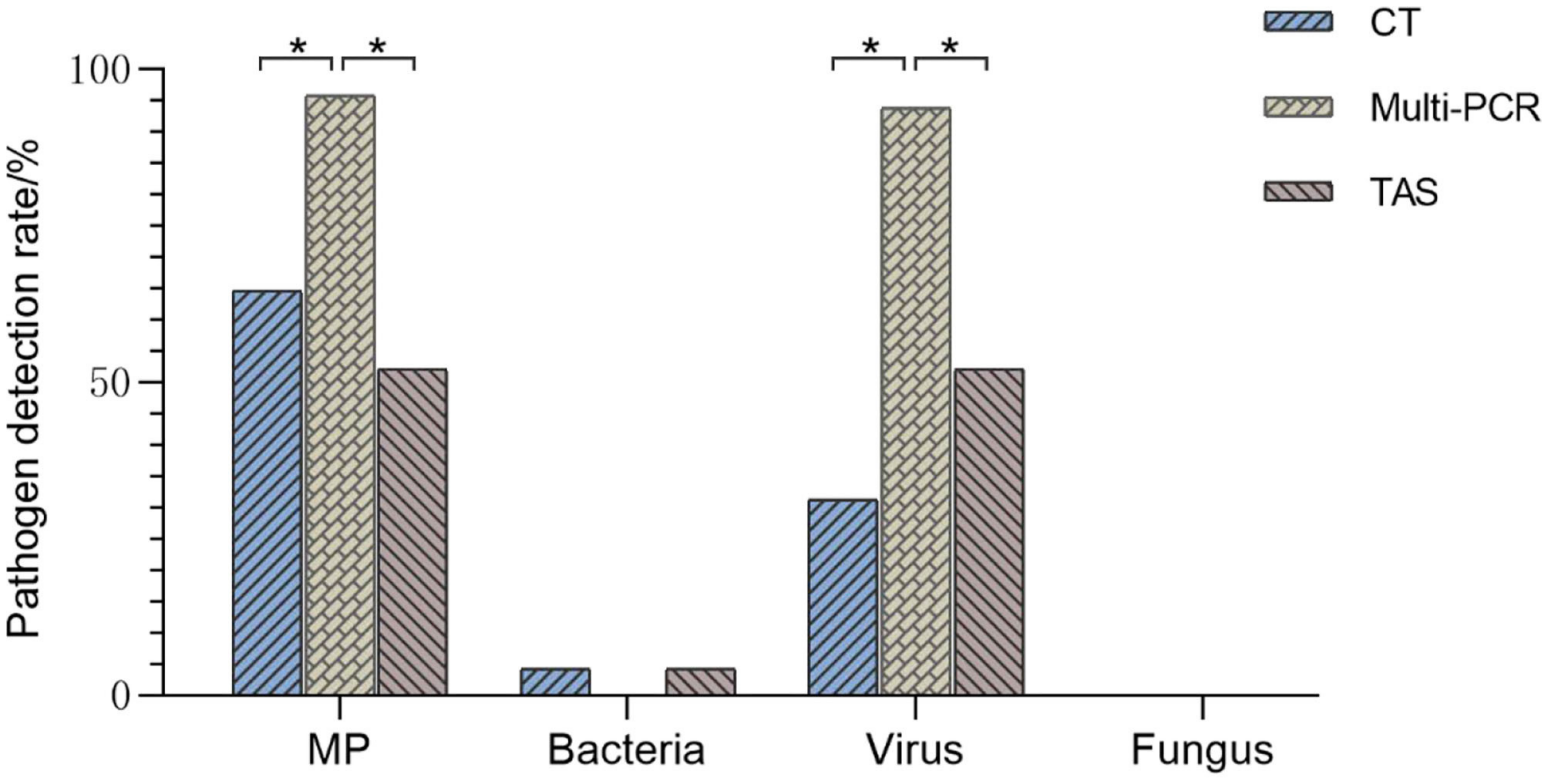
Comparison of pathogens detection results of three methods (TAS pathogen detection based on CT or Muti-PCR. *p < 0.01).

The positive rates of MP, ADV, FLUA, and RSV were 52.0% (25/48), 29.1% (14/48), and 14.5% (7/48), respectively. Mixed infections identifying of TAS based on CT, Multi-PCR, CT or Multi-PCR were 8.3% (4/48), 25.0% (12/48), and 35.4% (17/48) (Figure 3). Co-detection rates of pathogens by CT and Multi-PCR were 22.9% (11/48) and 93.7% (45/48). The Multi-PCR assay showed higher sensitivity for detecting MP and viruses than CT and TAS (Figure 4).

### Performance of TAS Positive Pathogens Identification Based on ID Reading

The raw data of this high-throughput gene TAS assay showed the reads of every targeted gene. The microoganisms could not be directly judged as infection, colonization, and pollution. Two ID doctors read the sequencing data and clinical features to reach a consensus. The results of TAS pathogens detection with NO “Golden Standards” but based on ID Reading were showed in Table 4. MP and ADV were still the two dominant pathogens. Based on sequencing read, the TAS assay presented more microoganisms in the ALF. Most of them were co-detected. SP, *Acinetobacter baumannii* (Ab), Hi and *Hemophilus parainfluenzae* (Hpi) were found in almost 20% of the samples. Strains of Pae, *Moraxella catarrhalis* (MC), and *Klebsiella pneumoniae* (Kpn) were found in three samples. Meanwhile, *Enterococcus, Corynebacterium striatum* (C.striatum), *Aerococcus viridans* (A.viridans), *Actinomyces naeslundii* (AN), and *Streptococcus* were detected in most of the ALF samples, which of the microoganisms were part of the normal flora of the upper respiratory tract and mouth in human beings. All Epstein-Barr virus (EBV) and Cytomegalovirus (CMV) were co-detected with other pathogens. Most of the Influenza virus were Type A (10 strains), and only one strain of Type B were found. Nine strains of cornavirus (OC43, 229E, HKU1, and NL63) were found in the 48 samples. No fungal or parasitic pathogens were identified in the specimens (Supplementary Material 5).

**Table 4:** TAS pathogens detection judged by ID Reading in 48 samples.

**Pathogens**	**Positive rate% (*n*)**	**Co-detection % (*n*)**	**Read range**
MP	52.0 (25)	100.0 (25)	4–24,945
ADV	31.2 (15)	100.0 (15)	7–6,574
SP	29.1 (14)	100.0 (14)	1–3,763
Ab	29.1 (14)	100.0 (14)	1–238
FLUA	20.8 (10)	100.0 (10)	23–9,456
hMPV	16.6 (8)	100.0 (8)	2–10,956
EBV	16.6 (8)	100.0 (8)	14–6,528
Hi	12.5 (6)	100.0 (6)	8–360
Hpi	12.5 (6)	100.0 (6)	5–52
OC43	10.4 (5)	100.0 (5)	7–6,716
RSV	10.4 (5)	100.0 (5)	1–1,083
CMV	8.3 (4)	100.0 (4)	1–1,573
HBov	4.1 (3)	100.0 (3)	3–13
HSV7	4.1 (3)	100.0 (3)	1–4
229E	4.1 (2)	100.0 (2)	6–18
PIV3	4.1 (2)	100.0 (2)	1–7,174
Pae	2.0 (1)	100.0 (1)	27,926
Kpn	2.0 (1)	100.0 (1)	5
MC	2.0 (1)	100.0 (1)	39
PIV1	2.0 (1)	100.0 (1)	48
FLUB	2.0 (1)	100.0 (1)	63
HKU1	2.0 (1)	100.0 (1)	1
NL63	2.0 (1)	100.0 (1)	2
# *Enterococcus*	62.5 (30)	/	2–1,957
#C.striatum	60.4 (29)	/	1–939
#A.viridans	54.1 (26)	/	1–156
#AN	31.2 (15)	/	1–114
#*Streptococcus*	31.2 (15)	/	1–3,485

*#Microganisms were part of normal flora of upper respiratory tract and mouth in human being or been judged as colonized bacteria*.

## Discussion

TAS and mNGS were believed two hopeful “future techniques” for the diagnosis of suspected pathogens from clinical samples (13). Based on next-generation sequencing technology, TAS and mNGS have effectively transformed infectious diseases research and expanded clinical understanding of infectious diseases or microbiome throughout the past decade (10, 14). TAS is better than mGNS by avoiding the interference of host DNA on the detection effectiveness, and can be used for low biomass samples (e.g., ALF/CSF). Our study attempted to rely on TAS reads to determine the results of pathogens detection in ALF samples (7, 11, 13, 15). Here we successively used CT and Multi-PCR as the Golden Standards to judge the results of TAS, to analyze the ability of TAS to detect pathogens. But we found that even very low TAS reads (Table 2) must be accepted as a positive result to “flatter” the results of CT or Multi-PCR and achieve the maximum positive coincidence rate.

The total pathogens detection rate by CT was 81.2% (39/48) in the 48 specimens, which included DFA, singleplex PCR, and culture, suggesting that there were indeed abundant possible respiratory pathogens in those samples, consistent with our previous research and other scholars' reports (7, 9, 16, 17). With the criteria for a positive TAS result both identified by TAS and CT, the total pathogens detection rate of TAS was 70.8% (34/48); there were no significant differences of total pathogens detection rate between TAS and CT (*p* = 0.232). When Multi-PCR was used as the only criterion to judge TAS results, we found that the detection rate of total pathogens in TAS (83.3%, 41/48) was lower than that of Multi-PCR (*p* = 0.003). When a positive TAS result was based on any of the other two assay positive reports, the total pathogens detection rate of TAS was up to 85.4% (41/48), and four CT negative samples were found to have positive pathogens (ADV, FLUA, MP, and MP+ADV). Even in the 48 samples that got positive results by Multi-PCR method, three of them (each one of RSV, hMPV, and Pae) were found missed by the Multi-PCR assay. Those results suggest that TAS is not inferior to CT in overall pathogens detection.

Mixed pathogens that coexisted were common in those 48 samples, proved by the three methods (Figure 3). Such results were not surprising (2, 7). MP and ADV were the two most frequently detected pathogens in all three assays, and also the most common pathogens of co-infection (18–20). TAS and Multi-PCR assay exhibited apparent advantages in identifying mixed infection than CT as reported in other articles (7, 9). We tried to show the possible “true” results of pathogens detection by TAS, instead of referring to the results of CT and Multi-PCR, and the raw data were judged again based on clinical information. We found all specimens had at least one possible respiratory pathogen, such as the reports in the Multi-PCR assay. High frequency of co-infections was found between viruses and bacteria (including MP), so as within different viruses. Herpes viruses (including EBV, CMV, and HSV7), HBov, and coronaviruses (including OC43, 229E, NL63, and HKU1) all showed as co-infection or bypassing pathogens.

Our previous research confirmed that viruses were the most prevalent etiological agents in hospitalized children with SARI in either Shanghai or Beijing, China (21). Eight common respiratory viruses (RSV, ADV, FLUA/B, PIV1-3, and hMPV) were found in 31.2% (15/48) of the 48 samples by DFA, while those common respiratory viruses were found in 45.8.9% (22/48) samples by the Multi-PCR assay in this study. The positive rates of ADV and FLUA were up to 29.1% (14/48) and 14.5% (7/48) in the TAS assay based on CT or Multi-PCR results. However, in general, the positive rate of those common respiratory viruses was lower than previous studies (21), which might relate to case selection in this study. When TAS was based on CT or Multi-PCR, TAS found four positive results in the CT-negative samples and three additional pathogens in the Multi-PCR positive samples. Such results demonstrated the unique advantages of this TAS assay.

This TAS assay also performed very well in the simultaneous detection of bacteria. Sp, Hi, and Hpi, considered the most common respiratory bacteria, were detected in a large proportion of the samples. Each strain of Pae, MC, and Kpn were found in three samples by TAS. The ability of the TAS assay to sensitively detect common lung bacteria was reassuring and encouraging. We noted 14 strains of Ab were positive in TAS, which suggested that those 47 chosen cases might have long time or repeated exposure in medical institutions due to their complex pulmonary infection, leading to the possibility of secondary nosocomial infection in some cases. Meanwhile, *Enterococcus, Corynebacterium striatum, A. viridans*, AN, and *Streptococcus*, part of the normal flora of the respiratory tract and mouth, also were detected from some of those ALF samples. That was suggesting some of the samples were contaminated by oropharyngeal secretions during the process of collecting or storage. The high frequency of opportunistic pathogens that appeared in the reports would interfere with the clinician judgment. For example, in sample 41, TAS results presented with 68 reads of MP, 142 reads of SP, seven reads ADV, and 98 reads of *Neisseria lactamica*. We believed that the *Neisseria lactamica* was the contaminated microorganism. We thought the reason why patients with severe pneumonia were mainly caused by MP and ADV in this study, different from the other reports, in which bacteria including *Pneumococcus, Streptococcus A*, and staphylococcus aureus were more common in severe pneumonia, might come from the samples collecting (22, 23).

This was a study with a small sample size, non-randomly selected cases, and single-center and retrospective research. Most of those SCAP patients had atelectasis or consolidation. We know that pathogens nucleic acids can remain at the site of infection for a long period, so detection of nucleic acids might not indicate the pathogens were still causing infections and requires treatments. We couldn't differentiate co-lonizations from infections by nucleic acid testing, including possible colonization of MP. Those deficiencies limited the broad representativeness of our results. In addition, we were forced to accept some very low TAS reads which might lead to a bias. In sample 39, the reads of MP in the TAS assay was only four. We could only accept such low reads as a positive result, in reference to both CT and Multi-PCR, indicating the presence of MP in that sample.

Overall, this TAS assay has improved the performance of detecting bacteriological and viral pathogens from ALF than CT. We anticipate that in the coming decade, a high-throughput gene TAS assay will be implemented in routine usage in clinical microbiology laboratories.

## Data Availability Statement

The original contributions presented in the study are included in the article/Supplementary Materials, further inquiries can be directed to the corresponding author/s.

## Ethics Statement

The studies involving human participants were reviewed and approved by Ethics Committee of pediatric hospital affiliated to Fudan University Application Institutions: children's hospital of Fudan University. Written informed consent from the participants' legal guardian/next of kin was not required to participate in this study in accordance with the national legislation and the institutional requirements.

## Author Contributions

JS, WT, RL, and LW conceived the experiments and analyzed the results. FL, YW, and YZ conducted the experiments, analyzed the results, and wrote the manuscript. FL collected clinical data. PS and QZ analyzed the results. LC, LS, and FL collected specimens and conducted the experiments. All authors reviewed the manuscript.

## Conflict of Interest

The authors declare that the research was conducted in the absence of any commercial or financial relationships that could be construed as a potential conflict of interest.

## Supplementary Material

Supplementary Material 1 Original clinical data of 47 cases.

Supplementary Material 2 RespiFinder 2SMART multiplex PCR kit.

Supplementary Material 3 High-throughput gene targeted amplicon sequencing assay.

Supplementary Material 4 Polygalacturonase (PG) library Agarose electrophoretogram and RNA library Agarose electrophoretogram of TAS.

Supplementary Material 5 TAS original data.
