# Supplementary material for: Etiology of Severe Pneumonia in Children in Alveolar Lavage Fluid Using a High-Throughput Gene Targeted Amplicon Sequencing Assay
Source: Front Pediatr. 2021 Jun 25;9:659164. doi: 10.3389/fped.2021.659164 (PMC8267249; doi:10.3389/fped.2021.659164)

Polygalacturonase(PG)library Agarose electrophoretogram and RNA library Agarose electrophoretogram of TAS.

PG library Agarose electrophoretogram

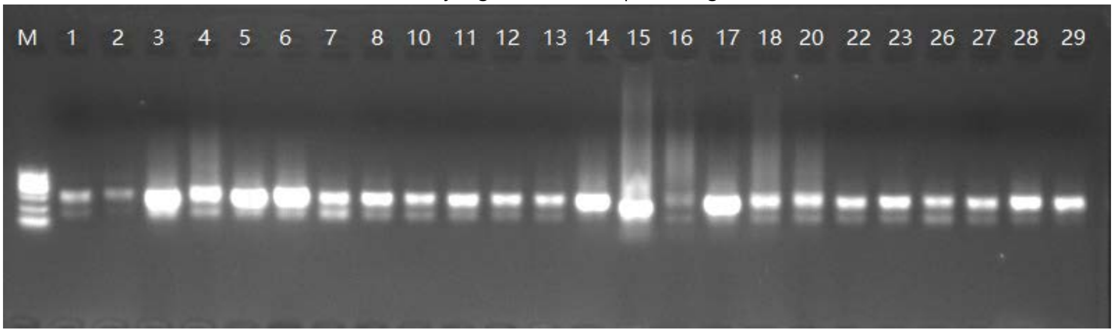

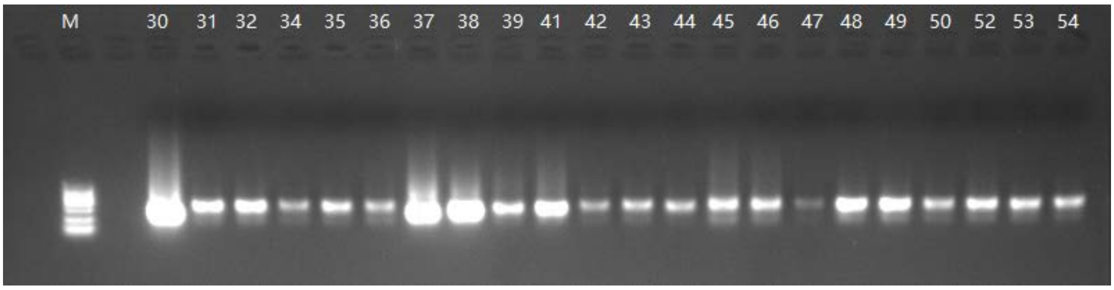

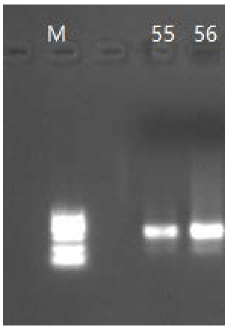

RNA library Agarose electrophoretogram

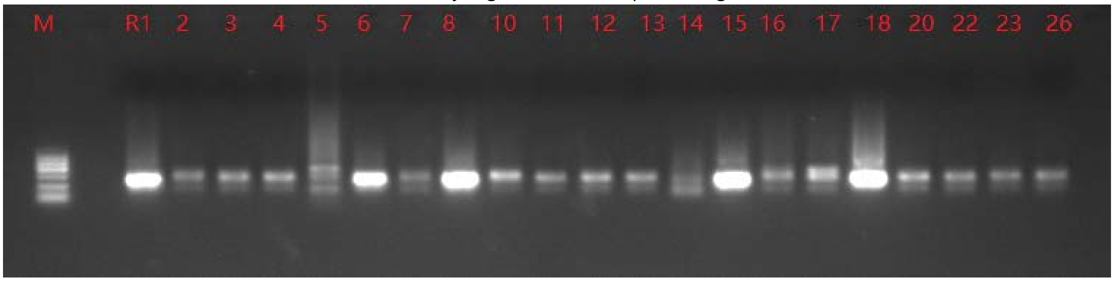

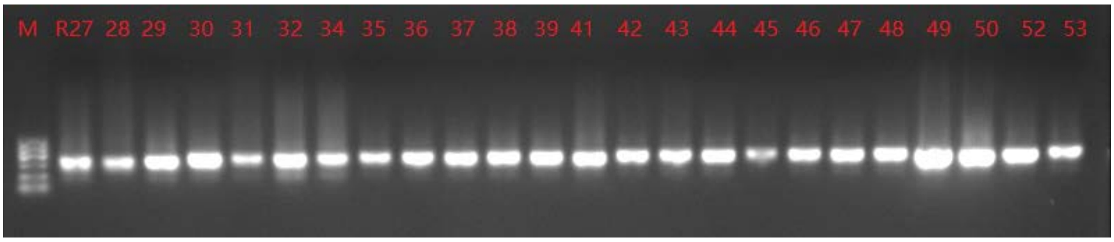

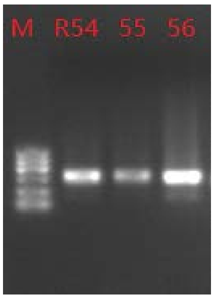

Supplement: Supplementary Material 5 — TAS original data. [file Data_Sheet_1.PDF]
